# An Overlapping Syndrome of Allergy and Immune Deficiency in Children

**DOI:** 10.1155/2012/658279

**Published:** 2011-09-12

**Authors:** Aleksandra Szczawinska-Poplonyk

**Affiliations:** Department of Pediatric Pneumonology, Allergology and Clinical Immunology, Poznan University of Medical Sciences, Szpitalna Street 27/33, 60-572 Poznan, Poland

## Abstract

Recurrent airway inflammations in children are an important clinical problem in pediatric practice. An essential challenge is differentiation between allergic background and immune deficiency, which is a difficult task taking into consideration individual predisposition to atopy, immune system maturation in the early childhood, as well as exposition to environmental allergens and microbial antigens. In this paper relationship between selected elements of innate and adaptive immunity, such as pattern-recognition receptors, complement components, dendritic cells, as well as immunoglobulins, and regulatory T lymph cells has been discussed. Particular attention has been paid to these mechanisms of the immune response which, depending on settings and timing of activation, predispose to allergy or contribute to tolerogenic phenotype. In the context of multifactorial conditioning of the innate and adaptive immunity governing the ultimate response and associations between allergy and immune deficiencies, these phenomena should be considered as pathogenetically not precluding, but as an overlapping syndrome.

## 1. Introduction

Development of the immune response in childhood is a dynamic process initiated within the fetal period and expanding in time through months and even years of child's life. Physiological phenomenon of immune system maturation, type and timing of activating allergens, and microbial antigens in conjunction with genetic predisposition to allergy are of crucial importance in determination of the proallergic or tolerogenic phenotype. Clinically, these considerations apply particularly to diagnostic and therapeutic dilemmas regarding recurrent airway inflammations in children, in which major questions concern differentiation between allergy and immune deficiency. Establishing a diagnosis is an essential challenge including common clinical manifestations, reciprocal impact of different clinical entities, overlapping pathomechanisms of allergic background, and defects of innate and adaptive immune responses, as well as deficiencies in factors playing a hitherto unexpected immunoregulatory role.

## 2. Maturation of the Immune System

Physiological phenomenon of maturation of the immune system, initiated within the fetal period, is dynamic in its character and is expanding in time through the first months and even years of child's life. Hence, within the neonatal period, infancy and early childhood dysfunction of numerous components of the immune system is observed.

Within the neonatal period, considerable immaturity characterizes the system of monocytes-macrophages. It consists in decreased expression of costimulatory molecules and diminished ability to differentiation into dendritic cells as well as weak production of IL-12 by monocytes [1]. Macrophages exhibit diminished response to IFN*γ*, decreased activity upon phagocytosis [1], and impairment of intracellular killing [2]. 

In neonates, the immaturity concerns function of dendritic cells. This consists in downregulated expression of costimulatory molecules by myeloid (mDC) and of plasmacytoid (pDC)dendritic cells, defective maturation and synthesis of cytokines—IFN*γ* and IL-12 as the response to signaling pathways downstream of Toll-like receptors engagement, particularly TLR4 and TLR9 and CD40 molecule as well as impaired ability to stimulate the immune response by pDC. The proposed mechanisms to explain the dysfunction of neonatal DC comprise intrinsic immaturity, defective interaction between dendritic cells and T lymphocytes as well as modulatory effect of natural regulatory T cells (nTreg). These cells, playing an important role during pregnancy and maintaining maternal tolerance to the fetus, are present in high numbers in neonates and are critical in maintaining homeostasis, immunological tolerance, and preventing autoimmunity. Neonatal nTregs exert their immunosuppressive function by the mechanism of interaction between molecules CTLA-4 and CD80/CD86 on antigen-presenting cells and by secretion of L-10 and TGF*β* [1].

Functional alterations of neonatal antigen-presenting cells may in turn lead to secondary defects of adaptive T-cell response. In neonates occurs a T-cell functional deficiency manifesting as downregulated expression of TCR/CD3 complex, adhesion molecules and CD40 ligand (CD40L, CD154), impaired cytotoxic activity of CD8+ T cells as well as decreased cytokine synthesis. Expression of a range of cytokines playing an essential role in the immune response, such as IL-4, IL-5, IFN*γ*, TNF*α*, and IL-12, is a dynamic process and their production increases with child's age [3]. Hodge et al. demonstrated a diminished number of neonatal T lymph cells and NK cells exhibiting expression of *β* chain of the IL-2 receptor. Moreover, the production level of cytokines such as IL-1*α*, IL-1*β*, and TNF*α* was lower compared to adults, pointing to decreased capacity to mount effective inflammatory response. On the contrary, the level and kinetics of expression of other functional molecules—CD71, HLA-DR; and CD152—were comparable to that in adults [4].

Predominance of the Th2-dependent immune response prevailing within the fetal period and expanding through the neonatal period and infancy [5–7] may be among others as a result of exerted activity of regulatory T cells, suppressing the proinflammatory Th1-mediated response [8]. Moreover, mechanisms of the innate immune response profiling development of the adaptive response towards advantageous Th1-or Th2-mediated immunity contribute to the predisposition or to the protection from asthma and allergy. Dose, settings, and timing of exposure to antigens are of crucial importance in modulating the immune response profile within the child's early life [9, 10].

Immaturity of the effector mechanisms and suppressive activity of the transplacentally transmitted maternal IgG antibodies contribute to the consequent deficiency of specific humoral response [5]. In neonates, rapid increase of the immunoglobulin M active in primary immune response to antigens, relatively high concentration of IgG of maternal origin and weak production of child's own immunoglobulins IgG and IgA manifest as dysgammaglobulinemia and reflect distinct dynamics of different isotype synthesis. In infants between the second and sixth months of life hypogammaglobulinemia continues as a result of still weak production of own and the breakdown of maternal immunoglobulin G. Delayed maturation of the humoral response manifests frequently as transient hypogammaglobulinemia of infancy (THI), which abates typically until the end of the second year of life [11], but may be prolonged even up to the fifth or sixth year of life [12] and hitherto the evaluation, if the immune defect in a child is transient or is signaling a permanent primary immune deficiency, may be difficult. In the recent study of Keles et al. [13], evaluating clinical and immunological features of THI children, asthma was the leading health problem present in 52% of patients.

## 3. Allergic Diseases Coexisting with Immune Deficiencies

### 3.1. Antibody Production Defects

To the clinical problem of concomitant occurrence of allergic diseases and primary immune deficiencies in children drew attention Klemola, who reported symptoms of atopic diseases in 50% of children with selective IgA deficiency (sIgAD) [14]. Interestingly, it was noted a better correlation between the prevalence and severity of the clinical course of allergic diseases in children within the first two years of life and a low normal IgA concentration in serum than a concentration of IgE increased above the normal value for age [15]. In the prospective study evaluating the same group of children at the age of four years, it was observed an association between occurrence of allergic diseases and asthma and decreased IgA and IgG4 subclass in serum as well as secretory salivary IgA [16]. The Papadopoulou study revealed not solely a higher prevalence of atopy in a group of children with selective IgA deficiency compared to a control group, but also pointed to the more frequent coexisting bronchial hyperreactivity and hypersensitivity to *Dermatophagoides pteronyssinus* in children with sIgAD [17]. The results of the study performed by Kutukculer et al. [18] indicated that partial IgA deficiency and IgG subclass deficiency are transient in 52% and 51% children, respectively, and that increases in serum immunoglobulins to age-related normal levels occur up to the sixth year of life. Exactly, in this group of children atopic diseases proved to occur more frequently than among children with complete selective IgA deficiency (in 41% and 24% patients, resp.). An analysis of correlation between clinical and immunological phenotypes was done in the recent report of Iranian authors [19] on the group of patients aged 4–32 years, showing IgA concentration below 62 mg/dl. Recurrent airway inflammations were the most frequent clinical problems, referring to 94% of patients. Allergic diseases-asthma, atopic dermatitis, allergic rhinitis, and conjunctivitis were diagnosed in 84% of patients, indicating the fact that not only a predisposition to infections resulting from an immunodeficiency solely, but also an allergic background considerably affects the clinical manifestation of the disease. Interestingly, an asthmatic phenotype was present exclusively in patients with selective IgA deficiency amounting 62% of the study group. On the contrary, in 38% of patients, who suffered from a complex immunodeficiency consisting in IgA deficiency, IgG subclass deficiency, deficiency of specific antibodies against polysaccharide antigens (sAbDs), and infections of the respiratory tract predominated as clinical manifestations and exclusively in this group of patients occurrence of bronchiectases was observed. Moraes et al. [20] in the study on the group of 41 severe asthmatic children showed an association between the degree of asthma control and recurrent airway infections as well as deficiencies within the immune system. In children with poor asthma control more frequently than in children with sufficiently controlled asthma (66% versus 55% of children) a deficiency of one or more IgG subclasses and IgG3 or IgG4 deficiency were diagnosed as well as solely in this group of children, a combined IgA and IgG subclass deficiency was revealed.

Single reports in the literature concerning selective immunoglobulin M deficiency (sIgMD) in pediatric population and in adults point to the recurrent respiratory tract infections as the predominant clinical feature. Allergic diseases and asthma coexist with this immune deficiency in 7-8% of affected children [21] and as much as 33% of adult patients [22].

Interestingly, an association between allergic diseases and immune deficiencies was observed in patients manifesting profound defects of antibody production. Shabestari et al. [23] reported a case of a boy with Bruton's agammaglobulinemia in whom concentrations of main classes of immunoglobulins were decreased within all isotypes including IgE (<0,1 IU/ml), B lymph cells bearing antigens CD19 and CD20 amounted less than 1% of normal value for age and the disease was confirmed by *BTK* gene mutation. Recurrent episodes of respiratory infections with obstruction of the lower airways were the most important clinical symptoms, and in additional diagnostic examinations, positive skin prick tests with alimentary and airborne allergens as well as bronchial hyperreactivity in spirometry were noted. A bias towards Th2-mediated immune response was also documented in patients with common variable immunodeficiency (CVID) through investigation of cytokine levels and demonstration of increased IL-4 and IL-10 production [24]. A high incidence of asthma, usually diagnosed after the initial presentation, was seen in 83% of pediatric patients with CVID as it was reported by Ogershok et al. [25].

### 3.2. Complement Deficiencies

Associations between allergic background of respiratory symptoms and immune defects are not exclusively confined to a dysregulation of immunoglobulin production and impaired balance between Th1 and Th2 lymph cells compartments. These phenomena are also determined by the activity of complement pathway components, establishing links between innate and adaptive immune responses. Although each pathway is activated by distinct pathway recognition receptors (PRRs), they all culminate in activation of C3 component, a central step in complement activation. Activation of C3 leads to the generation of anaphylatoxins C3a and C5a, which through binding their receptors on inflammatory cells proved to induce pathophysiological features of allergic inflammation. In support of this proallergic role of C3, its deficiency was shown on an animal model to have a protective effect against antigen-induced bronchial hyperreactivity [26, 27]. C5a, in turn, in allergic inflammation plays a dual role, both promoting and protective, depending on the inflammatory cells and cytokine environment upon its activation. Immunological role of C5a component consists principally in modulation of the adaptive immune response through altering the phenotype and function of antigen-presenting dendritic cells [28]. A deficiency in C5a leads to a shift of the proportion of the myeloid dendritic cells (mDCs) to plasmacytoid dendritic cells (pDCs), and to the consequent development of Th2-dependent effector phase. In this condition of a lack of C5a also an increase of the production of Th2-specific chemokines, CCL17 and CCL22 by pulmonary mDCs occurs, enhancing homing of Th2 lymph cells [29]. Impairment of the immune tolerance results from a defective stimulation of pDCs and absence of regulatory T-cell induction, corroborating a concept of a tolerogenic role of C5a and protective effect during allergen sensitization. A recognition of pattern-associated molecular patterns (PAMPs) leads to the activation of complement pathway by lectin proteins. A decreased level of a mannose-binding lectin (MBL), playing an important role in opsonization, was found in a group of asthmatic children [30]. Moreover, an association was shown between an allelic variant of *MBL2* gene leading to decreased MBL concentration in serum and higher risk of asthma in children presenting with recurrent and chronic *Chlamydia pneumonia* infection [31]. In adults an allelic MBL variant was not only associated with predisposing effect to asthma, but also correlated with a decrease in lung function [32].

Ficolins M, L, and H (1, 2, and 3, resp.), structurally similar to collectins, MBL, and surfactant protein A and D initiate the lectin pathway of complement activation through serin proteases MASPs [33]. Cedzynski et al. reported an association between relative L-ficolin deficiency and recurrent respiratory infections coexisting with asthma in children [34]. 

**Figure 1:**
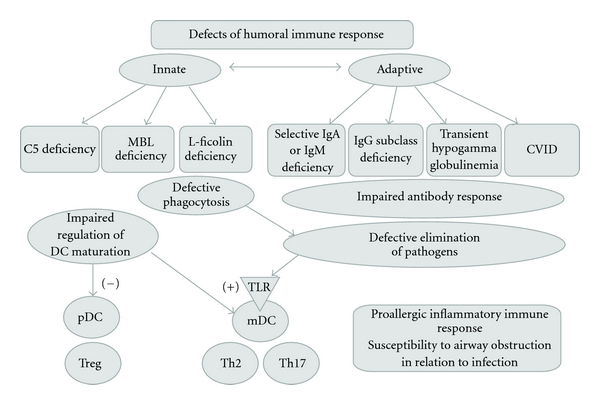
Humoral defects of innate and adaptive immune responses associated with proallergic effect.

Deficiencies of humoral innate and adaptive immune responses associated with proallergic effect are displayed on Figure 1.

### 3.3. Phagocyte Defect

Fitzpatrick et al. [35] demonstrated impaired alveolar macrophage phagocytosis in children with poorly controlled asthma. These findings suggest that a functional deficiency of innate immunity contributes to a defective antimicrobial response and recurrent airway inflammation resulting in exacerbations of asthma. This phenomenon of impaired phagocytosis may be explained by an alternative mechanism of macrophage activation potentiated by IL-4 in the Th2 microenvironment, where inhibition of phagocytosis associated with defective phagosome formation and concomitant increased proinflammatory cytokine secretion was observed by Varin et al. [36].

## 4. Role of Regulatory T Lymphocytes

**Figure 2:**
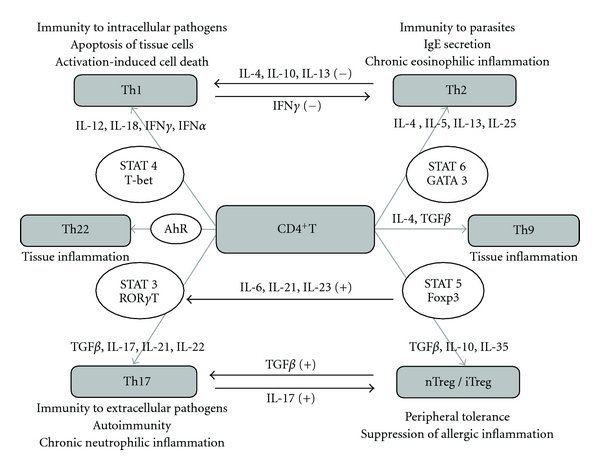
Mechanisms determining types of immune response and their mutual relationship as a result of transcription factors and cytokine activities towards CD4+ T cell subsets.

Natural Treg cells of a CD4+CD25+ phenotype are best characterized by an intracellular marker, a transcription factor Foxp3, playing an essential role in development and activity of these cells [37–40]. Inducible regulatory T lymph cells (iTreg, Tr1) arise as a result of activation of mature T cells in the settings lacking an optimal exposure to antigen or costimulation as well as in the environment of inhibitory cytokines and are characterized by IL-10 and TGF*β* production. Naïve CD4+ T cells may also develop into Tr1 cells in the presence of chronic stimulation with allergens infectious and tumor antigens. However, the suppressive function is not strictly Treg-specific and all CD4+ T cells exhibit suppressive activity in different degree. Mechanisms determining types of immune response and their mutual relationship as a result of transcription factors and cytokine activities towards CD4+ T cell subsets are displayed on Figure 2. 

For T regulatory lymphocytes, the following nonexclusive functions are proposed: prevention of autoimmunity by establishing and maintaining immunological tolerance to self-antigens, induction of maternal tolerance to the fetus, induction of tolerance against alimentary antigens, and suppression of pathogen-induced immunopathology [38, 39]. 

In allergic diseases and asthma, activation of CD4+ T cells plays a key role and allergic inflammation in the airways is mediated by subpopulations of effector Th2 and Th17 cells. Regulatory T cells, both nTregs having CD4+CD25+ phenotype and antigen-induced IL-10 secreting Tr1 cells achieve their regulatory effect by different pathways, inhibiting dendritic cells activity, suppressing effector Th2 and Th17 cells, suppressing mast cells and basophils, as well as decreasing migration of inflammatory cells to target tissues [41–44]. They also downregulate IgE synthesis and stimulate class switching towards anti-inflammatory isotypes—IgG4 subclass and, in a lesser extent, to IgA. Induction of IgA synthesis is first of all determined by activation of B lymphocytes through Toll-like receptors TLR9 and TLR7 pathway [45]. Hartl et al. [46] reported significantly decreased number of CD4+CD25+ T cells and lower concentration of cytokines IL-10 and TGF*β* in bronchoalveolar lavage fluid of asthmatic as compared to healthy children. Likewise, in patients manifesting symptoms of atopic diseases, Saito [47] demonstrated a smaller proportion of cells Foxp3+CD4+ than in asymptomatic individuals in the control group, having similar concentrations of IFN*γ* and IgE in serum as well as blood eosinophil count. These above-mentioned findings suggest that development of symptoms of allergic diseases is determined by mutual relationship between proinflammatory Th2 and Th17 lymph cells subsets and regulatory T cells. Recently, novel distinct populations of effector T helper cells involved in tissue inflammation have been demonstrated, namely Th9 cells, characterized by IL-9 and IL-10 secretion [48], and Th22 cells with IL-22 secretion and low expression levels of IL-17 [49, 50]. 

A deficiency of regulatory T cells resulting from *FOXP3* gene mutation is an essential factor in pathogenesis of IPEX syndrome (immune dysregulation, polyendocrinopathy, enteropathy, X-linked) [51]. In a range of primary immune deficiency diseases an immunological dysregulation may be consequent to functional regulatory T cells impairment and predominating Th2-dependent immune response, as it was demonstrated in patients with common variable immunodeficiency, a disorder predisposing to autoimmunity [52, 53]. A Th17 cells deficiency is a crucial immune abnormality in the hyperimmunoglobulin E syndrome (HIES), a complex immunodeficiency with a constellation of diverse clinical manifestations, unique predisposition to staphylococcal and mycotic infections, and heterogeneous genetic origin. In autosomal dominant hyper-IgE syndrome (AD-HIES) hypomorphic mutations in *STAT3* (signal transducer and activator of transcription) gene has been demonstrated [54], leading to impaired Th17 cells differentiation and defective multiple cytokine signaling, resulting in impaired upregulation of antimicrobial peptides [55]. Patients affected with autosomal recessive hyper-IgE syndrome (AR-HIES) due to DOCK8 (dedicator of cytokinesis 8) [56] or *Tyk2* (tyrosine kinase 2) gene mutation [57] share common features of HIES, such as elevated serum IgE concentration and eosinophilia as well as predisposition to staphylococcal and candidal infections however, different infection profile and clinical features suggest a distinct disease entity. 

Primary immune deficiencies of different genetic background associated with T-cell dysfunction and aberrant IgE production are displayed in Table 1.

## 5. Immunomodulatory Role of Pattern Recognition Receptors (PRRs)

Toll-like receptors (TLRs), along with retinoid acid-inducible gene-I-like receptors (RLRs) and nucleotide-binding oligomerization domain- (NODs) like receptors (NLR) are crucial elements of innate arm of immunity, recognizing pathogen-associated molecular patterns (PAMPs), and molecular structures specific for microbial pathogens. They are expressed in different cellular compartments, such as cell surface, endosome, lysosome or cytoplasm, and activate specific signaling pathways leading to expression of genes that tailor immune responses to particular microbes [58]. Toll-like receptors TLR1, TLR2, TLR4, TLR6, and TLR10 detect extracellular pathogen-associated signatures, while TLR3, TLR7, TLR8, and TLR9 recognize ligands derived from intracellular viral and bacterial pathogens. 

In B lymph cells, Toll-like receptors activation results in upregulation of activation markers, proliferation, cytokine secretion, terminal differentiation, and immunoglobulin secretion. It has been demonstrated that TLR9 stimulation of B cells with CpG motifs induces IgG class switch recombination (CSR). This effect results in inhibition of the IL-4 and CD40-dependent IgG1/IgE class switch and suppression of IgE production as well as simultaneous stimulation of IgA synthesis [59]. 

Extensive interactions and crosstalk among TLRs and other surface receptors is a characteristic feature of TLRs [60]. Both microbial antigens and allergens represent important trigger factors with effect on antigen-presenting cells and effector cells involved in allergic reactions. Therefore, it is likely that not separate but concomitant stimulation of both receptor types, namely, Toll-like receptors and high-affinity receptor for IgE (Fc*ε*RI) may occur under physiological conditions and in particular in the context of allergic and infectious diseases. Potential counterregulation and interaction of TLRs with IgE receptor-mediated immune response along with integrated signal from different receptor networks may therefore compose mechanisms which promote development of allergy [60]. 

Different TLRs, in particular TLR2 and TLR4 have been demonstrated to be expressed on mast cells, which can be activated to secrete diverse mediators and cytokines by IgE as well as specific antigens and products derived from either pathogens or the host during innate and adaptive immune responses [61, 62]. Depending on the type of ligand, TLR2 displays modulatory effect on Fc*ε*RI-mediated signaling and downregulation of Fc*ε*RI and attenuation of the IgE-dependent mast cell degranulation has been demonstrated in context to lipoteichoic acid [63]. These data point to the immunomodulatory function of mast cell TLR2 depending on the stimulating bacterial product. 

Interestingly, counterregulation of Fc*ε*RI, and TLR9 has been demonstrated on plasmacytoid dendritic cells. The capacity of pDC to release type 1 interferon (IFN-*α* and IFN-*β*) after TLR9 stimulation with specific ligands, unmethylated CpG motifs is substantially decreased after Fc*ε*RI aggregation and allergen challenge in vitro, indicating a cross talk also among these receptors, TLR9 and Fc*ε*RI [64]. Summarizing, costimulation of effector cells on the level of TLRs and Fc*ε*RI may lead to diverse ultimate effect, either protective or promoting allergic response and effective or impaired antimicrobial immune responses under specific conditions.

## 6. Effects of Vitamin D on Immune Functions

There is an emerging evidence of the role of vitamin D not only confined to calcium and bone homeostasis, but also to its pleiotropic and immunomodulatory effect on both innate and adaptive immune responses. The active form of vitamin D, 1,25(OH)_2_D binds to the vitamin D receptor (VDR), a nuclear receptor and ligand-activated transcription factor, expressed in many tissues and regulating cellular differentiation and function of many cell types, including the immune system. VDR expression is found, among others, in macrophages, dendritic cells, NK cells, T cells, and B cells. Upon activation, VDR elicits in these cells a potent anti-proliferative, prodifferentiative, and immunomodulatory effects on both transcription-dependent and transcription-independent actions [65]. Vitamin D inhibits the function of T lymphocytes both directly and via effects on antigen-presenting cells, particularly of Th1-associated cytokine production and IL-17 production by Th17 lymph cells involved in autoimmune and allergic processes, including asthma. The effect on Th2-mediated immune response is more complex and the reports point to both its inhibition and enhancement [66]. Vitamin D shows an inhibitory effect on dendritic cells by downregulating expression of costimulatory molecules CD40 and CD80/86, decreasing secretion of immunostimulatory IL-12. Concurrently, it leads to increased production of anti-inflammatory IL-10 by dendritic cells and CD4+CD25+ Treg cells. 

In the lungs airway, epithelial cells have been found to express high levels of 1alpha-hydroxylase, the vitamin D-activating enzyme. Calcitriol has also been shown to inhibit the synthesis and release of certain cytokines, such as RANTES (regulated on activation, normal T-cell expressed and secreted), PDGF (platelet-derived growth factor), and matrix metalloproteinases from bronchial smooth muscle cells, thereby leading to decreased inflammation and smooth muscle cell proliferation [67]. Additionally, on animal model, vitamin D receptors have been found in fetal type II alveolar epithelial cells, which may according to recent evidence play a role in the induction of regulatory T cells [68]. In several clinical studies, association of vitamin D with allergy and asthma has been investigated. In 2007, two simultaneously published reports [69, 70] have demonstrated higher risk of recurrent wheeze in children born from women with low vitamin D intake during pregnancy. Brehm et al. [71] showed a correlation between low levels of vitamin D and markers of asthma severity in children including hospitalizations, the use of medications, and airway hyperresponsiveness.

Therefore, vitamin D has been found to act as an immunomodulator relevant to pathomechanisms of antimicrobial response and allergy. In this context, vitamin D deficiency might be considered as an immunodeficiency state predisposing to airway infections and to decreased tolerogenic phenotype, with effect on complex interactions between genetic and environmental factors leading to development of asthma. 

## 7. Concluding Remarks

Multidirectional interactions and precise control of elements of the immune response determine homeostasis between effector mechanisms and tolerance. Above-mentioned associations between numerous elements of the immune system, the innate as well as adaptive immune response, and mechanisms predisposing to the development of allergy suggest complex considering of the clinical problem of recurrent airway inflammations in children. The above-mentioned findings point to the pathogenetic relationship between allergy and immune deficiencies which better than mechanisms of atopy correlate with symptomatology of the allergic diseases in children. These observations regarding mainly young children and infants presenting transient deficiencies of the immune response indicate a delayed maturation of immunological components as a phenomenon of crucial importance. A dysregulation of the immune response contributing to the defective antigen elimination in the early childhood of the predisposed individual may be considered as the critical risk factor preceding development of allergy. The immune deficiencies and allergy have mutual impact and are thus not precluding phenomena, but should be considered as a specific overlapping syndrome.

## Figures and Tables

**Table 1:** Primary immune Deficiencies and their genetic background, associated with T-cell dysfunction and aberrant IgE production.

Primary immune deficiencies with elevated IgE		
T-cell dysfunction	Immune deficiency	Genetic background
Treg cell deficiency	Immune dysregulation, polyendocrinopathy, enteropathy, X-linked (IPEX)	Forkhead box protein 3 (Foxp3) signal transducer and activator of transcription 5b (STAT5b)CD25
Treg cell dysfunction	Wiskott-Aldrich syndrome (WAS)	Wiskott-Aldrich syndrome protein (WASP)
Treg cell deficiency T-cell oligoclonality	Omenn syndrome (OS)	recombination activation genes (RAG1,RAG2) Artemis IL-7R zeta-associated protein, 70 kD (ZAP-70,) DNA ligase
Reduced NK cell cytotoxicityskewed Th1 phenotype	Comel-Netherton syndrome (CNS)	Serin protease inhibitor Kazal type (SPINK) lymphoepithelial Kazal type inhibitor (LEKTI)
T-cell oligoclonality	DiGeorge syndrome (DGS)—atypical complete form	Microdeletion 22q11
Reduced Th17 cells Treg cell dysfunctionmultiple cytokine signaling defect	Hyperimmunoglobulin E syndrome (HIES)	Signal transducer and activator of transcription 3 (STAT3) dedicator of cytokinesis 8 (DOCK8) tyrosine kinase 2 (Tyk2)
